# Predictive value of preoperative serum thyroglobulin and anti-thyroglobulin antibodies for central compartment lymph node metastasis in cN0 papillary thyroid carcinoma

**DOI:** 10.3389/fmolb.2026.1821840

**Published:** 2026-04-23

**Authors:** Xi Lin, Fangfang Wang, Xiaoliang Chen, Tianyao Yang

**Affiliations:** 1 Department of Thyroid and Breast Surgery, Tiantai People’s Hospital of Zhejiang Province (Tiantai Branch of Zhejiang Provincial People’s Hospital), Taizhou, Zhejiang, China; 2 Department of Thyroid, Breast & Hernia Surgery, Tiantai People’s Hospital of Zhejiang Province (Tiantai Branch of Zhejiang Provincial People’s Hospital), Taizhou, Zhejiang, China

**Keywords:** anti-thyroglobulin antibody, central lymph node metastasis, papillary thyroid carcinoma, predictive value, thyroglobulin

## Abstract

**Objective:**

To investigate the predictive value of preoperative serum thyroglobulin (Tg) and anti-thyroglobulin antibodies (TgAb) for central compartment lymph node metastasis (CLNM) in patients with clinically node-negative (cN0) papillary thyroid carcinoma (PTC).

**Methods:**

A retrospective analysis was conducted on clinical data from patients with cN0 PTC who underwent total thyroidectomy with central compartment lymph node dissection at our hospital between January 2018 and December 2023. Patients were randomly divided into a training cohort and a validation cohort in a 7:3 ratio. Data collected included patient demographics, clinicopathological characteristics, and preoperative serum Tg and TgAb levels. The outcome measure was pathologically confirmed CLNM status. Univariate and multivariate logistic regression analyses were employed to identify independent predictors of CLNM. The predictive value of preoperative serum Tg, TgAb, and the constructed prediction model for CLNM was assessed using receiver operating characteristic (ROC) curve analysis.

**Results:**

A total of 790 patients were enrolled in this study, comprising 553 in the training cohort and 237 in the validation cohort. The overall incidence of CLNM was 37.7% (298/790). In the training cohort, preoperative serum Tg and TgAb levels were significantly higher in the CLNM group compared to the non-CLNM group (*P* < 0.05). Multivariate logistic regression analysis identified age <45 years (OR = 1.675), maximal tumor diameter ≥1 cm (OR = 2.348), capsular invasion (OR = 2.757), preoperative serum Tg ≥ 34.7 ng/mL (OR = 2.257), preoperative serum TgAb ≥62.6 IU/mL (OR = 1.796), and log10 (Tg/TgAb +1) ≥ 0.21 (OR = 2.409) as independent predictors of CLNM (*P* < 0.05). The prediction model incorporating these six factors yielded AUCs of 0.863 and 0.819 for predicting CLNM in the training and validation cohorts, respectively.

**Conclusion:**

Preoperative serum Tg and TgAb are independent predictors of CLNM in cN0 PTC patients. The prediction model, which combined these serological markers with clinicopathological characteristics demonstrates good predictive performance and may offer valuable guidance for clinical decision-making regarding the necessity of prophylactic central compartment lymph node dissection in cN0 PTC patients.

## Introduction

Papillary thyroid carcinoma (PTC) is the most prevalent malignancy of the endocrine system, accounting for approximately 80%–85% of thyroid cancers, with its global incidence continuing to rise (Haugen et al., 2016; Rezkallah and Elsaify, 2022; Almajed et al., 2025). Although the overall prognosis for PTC patients is favorable, lymph node metastasis remains a critical factor influencing prognosis and recurrence. The central compartment is the most common site of lymph node metastasis in PTC. It is reported that among patients with clinically node-negative (cN0) PTC, the rate of pathologically confirmed central lymph node metastasis (CLNM) can be as high as 30%–50% (Taniuchi et al., 2024; Parvathareddy et al., 2022). Whether routine prophylactic central compartment lymph node dissection (pCND) should be performed for patients preoperatively assessed as cN0 remains a central controversy in clinical practice (Dobrinja et al., 2017).

The controversy surrounding pCND stems from conflicting evidence regarding its benefits versus risks. While some studies suggest that pCND may reduce locoregional recurrence and improve disease-free survival, others argue that it increases surgical complications without a significant improvement in overall survival. Unnecessary pCND carries substantial risks, including recurrent laryngeal nerve injury, which can lead to vocal cord paralysis, and damage to the parathyroid glands, potentially resulting in permanent hypoparathyroidism (Wang et al., 2023). The American Thyroid Association (ATA) guidelines currently recommend pCND primarily for high-risk tumors, while discouraging use in small solitary tumors due to lack of survival benefit and increased complication risks. The ATA guidelines highlighting the need for accurate preoperative risk stratification. Therefore, the identification of reliable preoperative predictors for CLNM is crucial for tailoring surgical strategies and preventing overtreatment. Accurately identifying high-risk subgroups for CLNM among cN0 PTC patients before surgery is essential for individualized treatment planning.

Currently, preoperative risk assessment for CLNM primarily relies on ultrasound characteristics, such as tumor size, location, margins, microcalcifications, and capsular invasion, along with clinicopathological factors including age and gender (Zhang et al., 2024; Qiao et al., 2024; Liang et al., 2025). Ultrasound is the main imaging modality for preoperative evaluation of cervical lymph node status in cN0 PTC patients. However, its accuracy is operator-dependent, and its sensitivity for assessing central compartment lymph nodes is limited, particularly for detecting micrometastases (Jin et al., 2020). In recent years, preoperative serological markers have garnered attention due to their non-invasive and easily accessible nature. Identifying reliable serological markers to predict CLNM holds significant clinical importance.

Thyroglobulin (Tg) is a specific protein synthesized and secreted by thyroid follicular epithelial cells (He et al., 2024). Under normal conditions, serum Tg levels are very low. In differentiated thyroid cancer, cancer cells retain the ability to synthesize and secrete Tg. Increased tumor burden, such as primary tumor growth or lymph node metastasis, may lead to elevated serum Tg levels (Kim et al., 2020). Recently, the value of preoperative serum Tg as a predictive marker has gained attention. A systematic review and meta-analysis indicated that preoperative serum Tg levels exceeding the upper limit of normal were associated with an increased risk of central lymph node metastasis in PTC patients (OR = 1.68) (Lu et al., 2024; Khan et al., 2025). Another study focusing on skip metastasis (lateral neck metastasis without central involvement) found that preoperative serum Tg ≤ 77 ng/mL was an independent risk factor (OR = 9.412) (Huang et al., 2020).

Anti-thyroglobulin antibodies (TgAb) are autoantibodies produced against Tg and are detected in approximately 10%–30% of PTC patients (Jia et al., 2020). TgAb not only interferes with the accurate measurement of serum Tg but may also be associated with tumor aggressiveness. Some studies suggest that TgAb positivity might be linked to an increased risk of lymph node metastasis in PTC (Vasileiadis et al., 2014). A study focusing on PTC patients with concomitant Hashimoto’s thyroiditis further identified preoperative serum TgAb levels >1150 IU/mL as an independent risk factor for predicting central lymph node metastasis (Min et al., 2021). However, the specific role and optimal cutoff value of TgAb in predicting CLNM in cN0 PTC remain insufficiently supported by evidence and are subject to debate.

Previous studies investigating Tg and TgAb as predictors of CLNM have several limitations. First, most studies included mixed populations of cN0 and cN+ patients, making it difficult to assess the predictive value specifically in cN0 cases. Second, many studies did not account for the potential confounding effect of autoimmune thyroid disease on TgAb levels. Third, the sample sizes in several studies were insufficient to develop robust predictive models. Fourth, few studies have explored the combined predictive value of Tg and TgAb ratios. These limitations highlight the need for well-designed studies focusing specifically on cN0 PTC patients.

The rationale for using the Tg/TgAb ratio as a predictive marker is based on several biological and clinical considerations. First, in patients with autoimmune thyroid disease, elevated TgAb may interfere with Tg measurement, leading to falsely low or undetectable Tg levels. The Tg/TgAb ratio accounts for this interference by normalizing Tg levels relative to TgAb concentrations. Second, the ratio may reflect the balance between thyroid tissue activity and autoimmune activity, which could be associated with tumor aggressiveness. Third, previous studies in differentiated thyroid cancer have shown that various Tg-based ratios, such as Tg/TSH ratio, may have superior prognostic value compared to Tg or TSH alone. The logarithmic transformation log10 (Tg/TgAb+1) was applied to normalize the distribution and handle cases where TgAb exceeds Tg levels.

Given the above background, the value of preoperative serum Tg and TgAb, alone or in combination, for predicting central lymph node metastasis in cN0 PTC requires further clarification through high-quality studies focused on specific populations. Therefore, this study aims to systematically evaluate the predictive value of preoperative serum Tg and TgAb for central lymph node metastasis in patients with cN0 PTC through a single-center retrospective cohort study, providing a scientific basis for clinical decision-making.

## Materials and methods

### Study population

This single-center retrospective cohort study was approved by the Ethics Committee of our hospital and conducted in strict accordance with the principles of the Declaration of Helsinki. Clinical data were retrospectively collected from patients with PTC who underwent initial surgical treatment at the Department of Thyroid Surgery of our hospital between January 2018 and December 2023.

Inclusion criteria: (1) Postoperative pathological diagnosis of classical PTC; (2) Preoperative high-resolution neck ultrasound assessment as cN0, meaning no lymph nodes meeting malignant criteria (based on size, shape, cortico-medullary structure, vascularity, or microcalcifications) were observed in the central or lateral neck compartments. Ultrasound assessments were performed independently by two sonographers with at least 5 years of experience; (3) Initial thyroid surgery; (4) Serum Tg and TgAb levels measured within 1 week before surgery; (5) Complete clinicopathological data and follow-up records.

Exclusion criteria: (1) Coexistence of other thyroid malignancies, such as medullary or anaplastic carcinoma; (2) History of thyroid or neck surgery; (3) Preoperative imaging or pathological evidence of lateral cervical lymph node metastasis (cN1b) or distant metastasis (M1); (4) Missing clinical or pathological data; (5) Preoperative thyroxine suppression therapy or use of medications affecting thyroid function.

**FIGURE 1:**
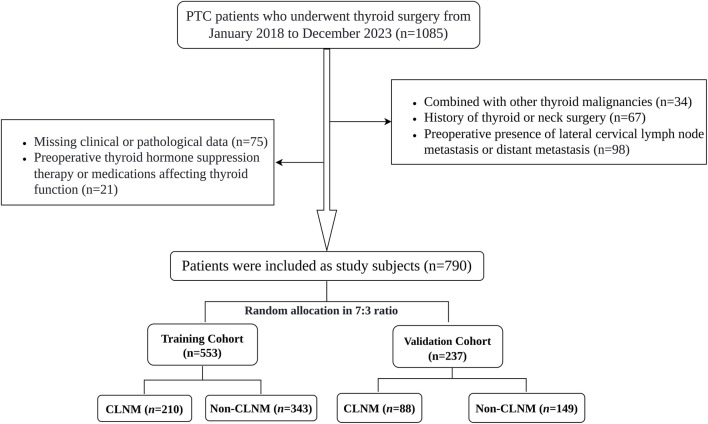
Flowchart of participants in this study.

A total of 1085 PTC patients were initially screened. After rigorous application of the inclusion and exclusion criteria, 790 patients with cN0 PTC were ultimately enrolled and randomly divided into a training cohort (n = 553) and a validation cohort (n = 237) in a 7:3 ratio. The patient selection and enrollment process is illustrated in Figure 1.

All patients underwent total thyroidectomy with prophylactic bilateral central compartment lymph node dissection. The indication for prophylactic CND was based on tumor characteristics and surgeon preference. No patients underwent therapeutic CND or unilateral CND. The surgical extent was consistent across all patients to ensure comparability.

### Data collection

The following data were collected from the hospital’s electronic medical record system:

1. Demographic characteristics: Age, sex, and body mass index (BMI);
2. Preoperative laboratory indicators: Preoperative serum Tg (ng/mL), TgAb (IU/mL), and TSH (mIU/L);
3. Preoperative imaging characteristics: Based on preoperative ultrasound reports, maximal tumor diameter, tumor location, multifocality, bilaterality, and capsular invasion were recorded;
4. Pathological characteristics: Including tumor size, histological subtype, capsular invasion, extrathyroidal extension (ETE), lymphovascular invasion (LVI), multifocality, and central lymph node metastasis status.

Clinical and ultrasound variables: Preoperative ultrasound features were re-reviewed and recorded by two sonographers: maximal tumor diameter (largest of three dimensions), location (upper, middle, lower pole, isthmus), number (unifocal/multifocal), internal echogenicity (hypoechoic/markedly hypoechoic/isoechoic/hyperechoic), microcalcification (present/absent), and invasion of or contact with the anterior thyroid capsule.

Serological markers: Preoperative serum Tg and TgAb were measured using electrochemiluminescence immunoassay (Roche Cobas e601). The Tg detection range was 0.04–500 ng/mL, with a functional sensitivity of 0.1 ng/mL. The positive threshold for TgAb was 115 IU/mL. The ratio log10 (Tg/TgAb +1) was calculated.

Pathological results: All surgical specimens were processed according to standard protocols. CLNM status was confirmed by two senior pathologists based on postoperative paraffin-embedded pathology results. The primary outcome was pathologically confirmed positive ipsilateral CLNM.

### Preoperative serum Tg and TgAb measurement

Fasting venous blood samples (5 mL) were collected from all cN0 PTC patients within 1 week before surgery between 7:00 and 9:00 a.m. Blood samples were left to stand at room temperature for 30 min, then centrifuged at 3,000 r/min for 10 min. Serum was separated, aliquoted, and stored in a −80 °C freezer until analysis, avoiding repeated freeze-thaw cycles. Thyroglobulin (Tg) assay: Serum Tg levels were measured using the Roche Cobas series automatic immunoassay analyzer via electrochemiluminescence immunoassay (ECLIA). TgAb status was simultaneously assessed during measurement to rule out potential interference with Tg measurement. Anti-thyroglobulin antibody (TgAb) assay: Serum TgAb levels were measured using an automatic immunoassay analyzer via chemiluminescence immunoassay (CLIA). All assays were performed strictly following the reagent manufacturer’s instructions and the laboratory’s standard operating procedures.

### Statistical analysis

Statistical analysis was performed using SPSS 26.0 and R software. The normality of continuous data was assessed using the Shapiro-Wilk test. Data following a normal distribution were expressed as mean ± standard deviation, and comparisons between groups were made using the independent samples t-test. Data not following a normal distribution were expressed as median (interquartile range), and comparisons between groups were made using the Mann-Whitney U test. Categorical data were expressed as frequencies (percentages), and comparisons between groups were made using the Chi-square test or Fisher’s exact test. In the training cohort, univariate logistic regression analysis was used to screen for potential predictors associated with CLNM. Variables with *P* < 0.05 in the univariate analysis were included in the multivariate logistic regression analysis to identify independent predictors of CLNM. Odds ratios (OR) and their 95% confidence intervals (CI) were calculated. A prediction model was constructed based on the independent predictors. Receiver operating characteristic (ROC) curve analysis was used to evaluate the predictive performance of preoperative serum Tg, TgAb, and the prediction model. The area under the curve (AUC) with its 95% CI, optimal cutoff values, sensitivity, and specificity were calculated. The predictors identified in the training cohort and their regression coefficients were applied to the validation cohort. The predicted risk score for each patient in the validation cohort was calculated, and the AUC of the model in this cohort was assessed. All hypothesis tests were two-sided, and *P*-value <0.05 was considered statistically significant.

## Results

### Baseline characteristics of patients

This single-center retrospective cohort study included 790 patients with cN0 PTC who underwent total thyroidectomy and central compartment lymph node dissection at our hospital between January 2018 and December 2023. The demographic characteristics, clinicopathological features, preoperative serum marker levels, and CLNM occurrence of the study population are detailed in Table 1. The mean age of the study population was 45.7 ± 12.7 years, with a female predominance (72.3%, 571/790). The mean BMI was 23.9 ± 3.3 kg/m^2^, and 48.7% of patients had a BMI ≥25 kg/m^2^. The overall incidence of CLNM was 37.7% (298/790) (Table 1). Patients were randomly divided into a training cohort (n = 553) and a validation cohort (n = 237) in a 7:3 ratio. There were no statistically significant differences in baseline characteristics between the training and validation cohorts (*P* > 0.05).

**TABLE 1:** Comparison of baseline characteristics between training cohort (*n* = 553) and validation cohort (*n* = 237) cN0 PTC patients.

Characteristics	Overall (n = 790)	Training cohort (n = 553)	Validation cohort (n = 237)	*P*
Demographic characteristics				
Age (years, mean ± SD)	45.7 ± 12.7	45.4 ± 12.6	45.8 ± 13.2	0.793
Age distribution				0.913
Age<45 years old, n (%)	369 (46.7%)	259 (46.8%)	110 (46.4%)	
Age ≥45 years old, n (%)	421 (53.3%)	294 (53.2%)	127 (53.6%)	
Gender				0.903
Male, n (%)	219 (27.7%)	154 (27.8%)	65 (27.4%)	
Female, n (%)	571 (72.3%)	399 (72.2%)	172 (72.6%)	
BMI(kg/m^2^, mean ± SD)	23.9 ± 3.3	23.9 ± 3.4	24.0 ± 3.3	0.899
BMI distribution				0.938
BMI≥25, n (%)	385 (48.7%)	270 (48.8%)	115 (48.5%)	
BMI<25, n (%)	405 (51.3%)	283 (51.2%)	122 (51.5%)	
Clinical pathological characteristics				
Maximum tumor diameter (cm, mean ± SD)	1.3 ± 0.6	1.3 ± 0.8	1.2 ± 0.6	0.859
Distribution of maximum tumor diameter				0.963
Maximum tumor diameter <1 cm, n (%)	349 (44.2%)	244 (44.1%)	105 (44.3%)	
Maximum tumor diameter ≥1 cm, n (%)	441 (55.8%)	309 (55.9%)	132 (55.7%)	
Tumor location (middle/lower third of the thyroid lobes), n (%)	516 (65.3%)	361 (65.3%)	155 (65.4%)	0.974
Multiple lesions, n (%)				1.000
Yes	260 (32.9%)	182 (32.9%)	78 (32.9%)	
No	530 (67.1%)	371 (67.1%)	159 (67.1%)	
Bilateral tumors, n (%)				1.000
Yes	140 (17.7%)	98 (17.7%)	42 (17.7%)	
No	650 (82.3%)	455 (82.3%)	195 (82.3%)	
Hypoechogenicity, n (%)				0.967
Yes	654 (82.8%)	458 (82.8%)	196 (82.7%)	
No	136 (17.2%)	95 (17.2%)	41 (17.3%)	
Microcalcification, n (%)				0.975
Yes	416 (52.7%)	291 (52.6%)	125 (52.7%)	
No	374 (47.3%)	262 (47.4%)	112 (47.3%)	
Capsular invasion, n (%)				1.000
Yes	260 (32.9%)	182 (32.9%)	78 (32.9%)	
No	530 (67.1%)	371 (67.1%)	159 (67.1%)	
Extrathyroidal invasion, n (%)				0.826
Yes	9 (1.1%)	6 (1.1%)	3 (1.3%)	
No	781 (98.9%)	547 (98.9%)	234 (98.7%)	
Hashimoto’s thyroiditis, n (%)				0.985
Yes	173 (21.9%)	121 (21.9%)	52 (21.9%)	
No	617 (78.1%)	432 (78.1%)	185 (78.1%)	
Preoperative serum biomarkers				
Tg [ng/mL, median (IQR)]	33.1 (13.3, 85.4)	32.9 (13.4, 85.3)	33.1 (13.7, 86.0)	0.854
TgAb [IU/mL, median (IQR)]	55.9 (22.6, 176.2)	55.8 (22.5, 176.2)	56.0 (22.5, 176.0)	0.869
log10 (Tg/TgAb +1) [median (IQR)]	0.20 (0.08, 0.42)	0.20 (0.08, 0.42)	0.19 (0.07, 0.42)	0.903
TSH(mIU/L, mean ± SD)	2.1 ± 1.2	2.1 ± 1.2	2.0 ± 1.0	0.889
**CLNM, n (%)**	298 (37.7%)	210 (38.0%)	88 (37.1%)	0.823

BMI, body mass index; Tg, thyroglobulin; TgAb, anti-thyroglobulin antibody; TSH, thyroid-stimulating hormone; CLNM, central lymph node metastasis. The *P* value represents the comparison between the Training Cohort and the Validation Cohort.

### Comparison between CLNM and non-CLNM groups

Based on postoperative pathological results, in the training cohort (n = 553), 210 patients (38.0%) were in the central lymph node metastasis (CLNM) group, and 343 patients (62.0%) were in the non-CLNM group. A comparison of baseline characteristics between the CLNM and non-CLNM groups in the training cohort is shown in Table 2.

**TABLE 2:** Comparison of baseline characteristics between cN0 PTC patients with and without CLNM in the training cohort (*n* = 553).

Characteristics	Overall (*n* = 553)	CLNM group (*n* = 210)	Non-CLNM group (*n* = 343)	*P*
Demographic characteristics				
Age (years, mean ± SD)	45.4 ± 12.6	41.5 ± 10.8	47.6 ± 12.3	0.027
Age distribution				0.001
Age<45 years old, n (%)	259 (46.8%)	117 (55.7%)	142 (41.4%)	
Age ≥45 years old, n (%)	294 (53.2%)	93 (44.3%)	201 (58.6%)	
Gender				<0.001
Male, n (%)	154 (27.8%)	79 (37.6%)	75 (21.9%)	
Female, n (%)	399 (72.2%)	131 (62.4%)	268 (78.1%)	
BMI(kg/m^2^, mean ± SD)	23.9 ± 3.4	24.3 ± 3.9	23.4 ± 3.2	0.329
BMI distribution				0.138
BMI≥25, n (%)	270 (48.8%)	111 (52.9%)	159 (46.4%)	
BMI<25, n (%)	283 (51.2%)	99 (47.1%)	184 (53.6%)	
Clinical pathological characteristics				
Maximum tumor diameter (cm, mean ± SD)	1.3 ± 0.8	1.5 ± 0.7	1.1 ± 0.6	0.009
Distribution of maximum tumor diameter				<0.001
Maximum tumor diameter <1 cm, n (%)	244 (44.1%)	68 (32.4%)	176 (51.3%)	
Maximum tumor diameter ≥1 cm, n (%)	309 (55.9%)	142 (67.6%)	167 (48.7%)	
Tumor location (middle/lower third of the thyroid lobes), n (%)	361 (65.3%)	153 (72.9%)	208 (60.6%)	0.003
Multiple lesions, n (%)				<0.001
Yes	182 (32.9%)	92 (43.8%)	90 (26.2%)	
No	371 (67.1%)	118 (56.2%)	253 (73.8%)	
Bilateral tumors, n (%)				0.385
Yes	98 (17.7%)	41 (19.5%)	57 (16.6%)	
No	455 (82.3%)	169 (80.5%)	286 (83.4%)	
Hypoechogenicity, n (%)				0.344
Yes	458 (82.8%)	178 (84.8%)	280 (81.6%)	
No	95 (17.2%)	32 (15.2%)	63 (18.4%)	
Microcalcification, n (%)				<0.001
Yes	291 (52.6%)	153 (72.9%)	138 (40.2%)	
No	262 (47.4%)	57 (27.1%)	205 (59.8%)	
Capsular invasion, n (%)				<0.001
Yes	182 (32.9%)	96 (45.7%)	86 (25.1%)	
No	371 (67.1%)	114 (54.3%)	257 (74.9%)	
Extrathyroidal invasion, n (%)				0.542
Yes	6 (1.1%)	3 (1.4%)	3 (0.9%)	
No	547 (98.9%)	207 (98.6%)	340 (99.1%)	
Hashimoto’s thyroiditis, n (%)				0.841
Yes	121 (21.9%)	45 (21.4%)	76 (22.2%)	
No	432 (78.1%)	165 (78.6%)	267 (77.8%)	
Preoperative serum biomarkers				
Tg [ng/mL, median (IQR)]	32.9 (13.4, 85.3)	48.7 (19.5, 116.9)	27.5 (12.6, 67.8)	<0.001
TgAb [IU/mL, median (IQR)]	55.8 (22.5, 176.2)	67.6 (24.3, 180.9)	48.8 (19.8, 152.6)	<0.001
log10 (Tg/TgAb +1) [median (IQR)]	0.20 (0.08, 0.42)	0.24 (0.11, 0.52)	0.19 (0.08, 0.39)	<0.001
TSH(mIU/L, mean ± SD)	2.1 ± 1.2	2.4 ± 1.5	1.9 ± 1.3	0.023

CLNM, central lymph node metastasis; BMI, body mass index; Tg, thyroglobulin; TgAb, anti-thyroglobulin antibody; TSH, thyroid-stimulating hormone. The *P* value represents the comparison between the CLNM, group and the non-CLNM, group.

The CLNM group had a significantly younger mean age (41.5 ± 10.8 years vs. 47.6 ± 12.3 years, *P* = 0.027). The proportion of patients aged <45 years was significantly higher in the CLNM group (55.7%, 117/210) compared to the non-CLNM group (41.4%, 142/343). The proportion of males was significantly higher in the CLNM group (37.6% vs. 21.9%, *P* < 0.001).

The proportion of patients with a maximal tumor diameter ≥1 cm was significantly higher in the CLNM group (67.6% vs. 48.7%, *P* < 0.001). Tumors located in the middle/lower third of the thyroid lobes were more frequent in the CLNM group (72.9%, 153/210) than in the non-CLNM group (60.6%, 208/343) (*P* = 0.003). Multifocality was more common in the CLNM group (43.8% vs. 26.2%, *P* < 0.001). The CLNM group also had a higher proportion of microcalcifications (72.9% vs. 40.2%, *P* < 0.001).

Preoperative serum Tg and TgAb levels were significantly higher in the CLNM group compared to the non-CLNM group (both *P* < 0.001). The calculated ratio log10 (Tg/TgAb +1) was also significantly higher in the CLNM group (*P* < 0.001). Preoperative serum TSH levels were significantly higher in the CLNM group (*P* = 0.023).

### The predictive value of preoperative serum Tg and TgAb for CLNM in cN0 PTC patients

This study used ROC curve analysis to evaluate the predictive value of preoperative serum Tg, preoperative serum TgAb, and log10 (Tg/TgAb +1) for predicting CLNM in cN0 PTC patients.

**FIGURE 2:**
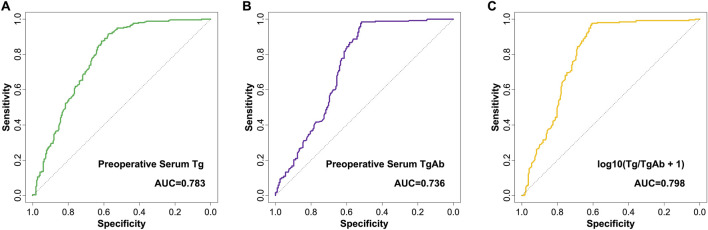
ROC curves of preoperative serum Tg **(A)**, preoperative serum TgAb **(B)**, and log10 (Tg/TgAb +1) **(C)** for predicting central compartment lymph node metastasis in cN0 papillary thyroid carcinoma.

ROC curve analysis revealed that the AUC for preoperative serum Tg in predicting CLNM in cN0 PTC was 0.783 (95% CI: 0.649–0.917), with a sensitivity of 84.9% and specificity of 72.8% (Figure 2A; Table 3). The AUC for preoperative serum TgAb in predicting CLNM in cN0 PTC was 0.736 (95% CI: 0.606–0.887), with a sensitivity of 82.3% and specificity of 70.9% (Figure 2B; Table 3). The AUC for log10 (Tg/TgAb +1) in predicting CLNM in cN0 PTC was 0.798 (95% CI: 0.669–0.932), with a sensitivity of 84.5% and specificity of 75.7% (Figure 2C; Table 3). Furthermore, ROC curve analysis revealed that the optimal cutoff values for predicting CLNM in cN0 PTC patients were 34.7 ng/mL for preoperative serum Tg, 62.6 IU/mL for preoperative serum TgAb, and 0.21 for the Tg/TgAb ratio log10 (Tg/TgAb +1) (Table 3).

**TABLE 3:** ROC curve analysis of preoperative serum Tg, preoperative serum TgAb, and log10 (Tg/TgAb +1) for predicting CLNM in cN0 PTC patients.

Item	AUC	95% CI	Sensitivity	Specificity	*P*	Cutoff value
Preoperative serum Tg (ng/mL)	0.783	0.649–0.917	84.9%	72.8%	<0.001	≥34.7
Preoperative serum TgAb (IU/mL)	0.736	0.606–0.887	82.3%	70.9%	0.001	≥62.6
log10 (Tg/TgAb +1)	0.798	0.669–0.932	84.5%	75.7%	<0.001	≥0.21

AUC, area under curve; 95% CI, 95% confidence interval; Tg, Thyroglobulin; TgAb, Anti-thyroglobulin antibody.

These ROC results indicate that preoperative serum Tg and log10 (Tg/TgAb +1) have good predictive ability for the CLNM in cN0 PTC patients. Preoperative serum Tg level, and log10 (Tg/TgAb +1) are useful for predicting CLNM.

### Analysis of factors predicting CLNM in cN0 PTC patients

**FIGURE 3:**
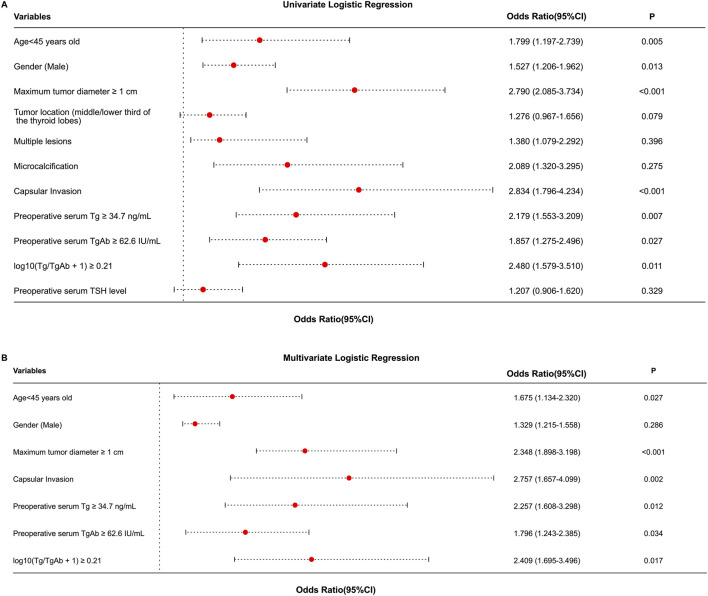
Forest plots of univariate **(A)** and multivariate **(B)** Logistic regression analysis of factors influencing CLNM in cN0 PTC patients.

Univariate regression analysis was performed on demographic, clinicopathological characteristics, and preoperative serological indicators of 553 patients with cN0 PTC in the training cohort to screen for potential factors associated with CLNM. The dependent variable was CLNM in cN0 PTC (1 = Metastasis, 0 = No), and the independent variables were clinical indicators showing significant differences (P < 0.05) between the CLNM and non-CLNM groups in Table 2. Univariate logistic regression analysis showed that Age <45 years old (OR = 1.799, 95% CI: 1.197–2.739), Gender (Male) (OR = 1.527, 95% CI: 1.206–1.962), Maximum tumor diameter ≥1 cm (OR = 2.790, 95% CI: 2.085–3.734), Capsular Invasion (OR = 2.834, 95% CI: 1.796–4.234), Preoperative serum Tg ≥ 34.7 ng/mL (OR = 2.179, 95% CI: 1.553–3.209), Preoperative serum TgAb ≥62.6 IU/mL (OR = 1.857, 95% CI: 1.275–2.496), and log10 (Tg/TgAb +1) ≥ 0.21 (OR = 2.480, 95% CI: 1.579–3.510) were significantly associated with CLNM (*P* < 0.05) (Table 4; Figure 3A).

**TABLE 4:** Univariate and multivariate logistic regression analysis of factors influencing CLNM in cN0 PTC.

Variables	Univariate logistic regression analysis		Multivariate logistic regression analysis	
	*OR* (95% CI)	*P*	*OR* (95% CI)	*P*
Age<45 years old	1.799 (1.197–2.739)	0.005	1.675 (1.134–2.320)	0.027
Gender (male)	1.527 (1.206–1.962)	0.013	1.329 (1.215–1.558)	0.286
Maximum tumor diameter ≥1 cm	2.790 (2.085–3.734)	<0.001	2.348 (1.898–3.198)	<0.001
Tumor location (middle/lower third of the thyroid lobes)	1.276 (0.967–1.656)	0.079	-	-
Multiple lesions	1.380 (1.079–2.292)	0.396	-	-
Microcalcification	2.089 (1.320–3.295)	0.275	-	-
Capsular invasion	2.834 (1.796–4.234)	<0.001	2.757 (1.657–4.099)	0.002
Preoperative serum Tg ≥ 34.7 ng/mL	2.179 (1.553–3.209)	0.007	2.257 (1.608–3.298)	0.012
Preoperative serum TgAb ≥62.6 IU/mL	1.857 (1.275–2.496)	0.027	1.796 (1.243–2.385)	0.034
log10 (Tg/TgAb +1) ≥ 0.21	2.480 (1.579–3.510)	0.011	2.409 (1.695–3.496)	0.017
Preoperative serum TSH level	1.207 (0.906–1.620)	0.329	-	-

OR, odds ratio; 95% CI, 95% Confidence Interval; Tg, Thyroglobulin; TgAb, Anti-thyroglobulin antibody; TSH, thyroid-stimulating hormone.

Variables with *P* < 0.05 in the univariate analysis were included in the multivariate logistic regression analysis to determine independent predictors of CLNM. Multivariate logistic regression analysis revealed that Age <45 years old (OR = 1.675, 95% CI: 1.134–2.320), Maximum tumor diameter ≥1 cm (OR = 2.348, 95% CI: 1.898–3.198), Capsular Invasion (OR = 2.757, 95% CI: 1.657–4.099), Preoperative serum Tg ≥ 34.7 ng/mL (OR = 2.257, 95% CI: 1.608–3.298), Preoperative serum TgAb ≥62.6 IU/mL (OR = 1.796, 95% CI: 1.243–2.385), and log10 (Tg/TgAb +1) ≥ 0.21 (OR = 2.409, 95% CI: 1.695–3.496) were independent predictors of CLNM (*P* < 0.05) (Table 4; Figure 3B).

Therefore, univariate and multivariate logistic regression analyses identified six factors as risk factors for CLNM in cN0 PTC patients: Age <45 years old, Maximum tumor diameter ≥1 cm, Capsular Invasion, Preoperative serum Tg ≥ 34.7 ng/mL, Preoperative serum TgAb ≥62.6 IU/mL, and log10 (Tg/TgAb +1) ≥ 0.21.

### The predictive performance of the model for CLNM in cN0 PTC patients

**FIGURE 4:**
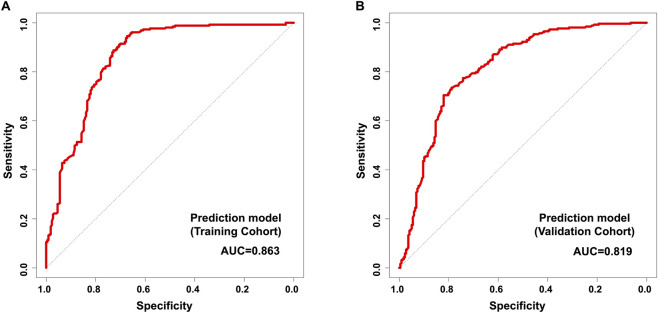
ROC curves of prediction model for predicting central compartment lymph node metastasis in training cohort **(A)** and validation cohort **(B)** cN0 PTC patients.

The prediction model, based on six independent predictors selected, encompasses both pathological characteristics and serological indicators. ROC curve analysis was used to evaluate the predictive performance of the model for CLNM in training cohort and validation cohort cN0 PTC patients. ROC curve analysis revealed that the AUC for prediction model in predicting CLNM in training cohort cN0 PTC was 0.863 (95% CI: 0.757–0.979), with a sensitivity of 88.9% and specificity of 73.6% (Figure 4A; Table 5). The AUC for prediction model in predicting CLNM in validation cohort cN0 PTC was 0.819 (95% CI: 0.679–0.955), with a sensitivity of 83.6% and specificity of 71.9% (Figure 4B; Table 5). These ROC results indicate that prediction model of this study has good predictive ability for the CLNM in cN0 PTC patients.

**TABLE 5:** ROC curve analysis of prediction model for predicting CLNM in training cohort and validation cohort cN0 PTC patients.

Item	AUC	95% CI	Sensitivity	Specificity	*P*
Prediction model (training cohort)	0.863	0.757–0.979	88.9%	73.6%	<0.001
Prediction model (validation cohort)	0.819	0.679–0.955	83.6%	71.9%	<0.001

AUC, area under curve; 95% CI, 95% confidence interval.

## Discussion

This single-center retrospective cohort study systematically evaluated the predictive value of preoperative serum Tg and TgAb for CLNM in patients with cN0 PTC and successfully constructed a prediction model incorporating these serological markers along with demographic and clinicopathological characteristics. The main findings of this study are as follows: In the training cohort, preoperative serum Tg and TgAb levels were significantly higher in the CLNM group compared to the non-CLNM group (*P* < 0.05). ROC curve analysis showed that the optimal cutoff value for preoperative serum Tg in predicting CLNM was 34.7 ng/mL (AUC = 0.783), for TgAb was 62.6 IU/mL (AUC = 0.736), and for log10 (Tg/TgAb+1) was 0.21 (AUC = 0.798). Multivariate logistic regression analysis identified age <45 years (OR = 1.675), maximal tumor diameter ≥1 cm (OR = 2.348), capsular invasion (OR = 2.757), preoperative serum Tg ≥ 34.7 ng/mL (OR = 2.257), preoperative serum TgAb ≥62.6 IU/mL (OR = 1.796), and log10 (Tg/TgAb+1) ≥ 0.21 (OR = 2.409) as independent predictors of CLNM (*P* < 0.05). The prediction model based on these six factors yielded AUCs of 0.863 and 0.819 for predicting CLNM in the training and validation cohorts, respectively.

This study confirms that preoperative serum Tg level is an independent predictor of CLNM, consistent with previous research findings (Huang et al., 2020). As a thyroid-specific protein, elevated Tg levels reflect the metabolic activity and tumor burden of thyroid cancer tissue. When lymph node metastasis is present, metastatic foci can also secrete Tg, leading to a further increase in serum Tg levels (Nangadda et al., 2025; Chang et al., 2022). Notably, this study is the first to systematically evaluate the value of the Tg/TgAb ratio in predicting CLNM. The results showed that log10 (Tg/TgAb+1) ≥ 0.21 was a strong predictor of CLNM (OR = 2.409), and its predictive efficacy was superior to that of Tg or TgAb alone. This may be because the Tg/TgAb ratio reflects the balance between tumor secretory activity (Tg) and the host immune response (TgAb), thereby providing a more comprehensive assessment of tumor biology.

Chang et al.'s study showed that preoperative serum Tg was an independent risk factor for cervical lymph node metastasis but did not evaluate TgAb or the Tg/TgAb ratio (Chang et al., 2022). Min et al. studied PTC patients with concomitant Hashimoto’s thyroiditis and found that high TgAb levels were a risk factor for CLNM, but the sample size was limited (n = 214) (Min et al., 2021). Compared with previous studies, the present study confirms these findings in a larger sample, providing higher statistical power, and introduces the novel concept of the Tg/TgAb ratio, incorporating log10 (Tg/TgAb+1) as a predictive indicator for the first time.

In this study, the finding that TgAb was an independent predictor of CLNM while HT was not may seem counterintuitive. Several explanations are possible. First, the presence of HT was determined histologically, which may not fully reflect the functional autoimmune activity. Second, TgAb levels may vary among HT patients, and only those with higher TgAb titers may have increased risk of CLNM. Third, the sample size may have been insufficient to detect the association between HT and CLNM. Fourth, TgAb may reflect tumor-induced immune responses rather than just autoimmune thyroid disease. This finding suggests that TgAb levels may be more informative than the binary presence or absence of HT. This highlights the complex interplay between tumor biology and immune responses in PTC patients.

Another noteworthy discrepancy lies between the standard laboratory positive threshold for TgAb (115 IU/mL) and the lower predictive cutoff of 62.6 IU/mL identified in this study for CLNM prediction. This finding suggests that even subclinical elevations in TgAb may be clinically significant in the context of CLNM risk assessment. The standard threshold is primarily used for diagnosing autoimmune thyroid disease, whereas the cutoff identified in this study is optimized specifically for predicting metastatic risk. This indicates that the clinical relevance of TgAb levels may differ between diagnostic and prognostic applications, underscoring the need for context-specific interpretation.

Regarding thyroid-stimulating hormone (TSH), the study revealed that the preoperative TSH level in the CLNM group was significantly higher than in the non-CLNM group (*p* = 0.023) in Table 2. However, in the univariate logistic regression analysis presented in Table 4, TSH as a predictor did not reach statistical significance (*p* = 0.329). This difference can be attributed to the distinct types of associations evaluated by these two statistical methods. The *p*-value in Table 2 reflects the mean difference of TSH level between CLNM group and non-CLNM group, indicating a statistical difference in TSH levels between these two groups. Conversely, the univariate logistic regression analysis in Table 4 assessed the predictive ability of TSH alone as an independent predictor for the occurrence of CLNM. Although a difference in TSH levels exists between the two groups, the pattern or magnitude of this difference might not be sufficient to demonstrate a statistically significant independent predictive effect within the logistic regression model. This suggests that while TSH levels may have a certain correlation with CLNM status, it is not enough as a predictor of CLNM.

The results of this study have important clinical implications. First, preoperative measurement of Tg and TgAb levels is simple, cost-effective, and non-invasive, making them suitable for routine screening in cN0 PTC patients. Second, the developed prediction model, incorporating these serological markers, can effectively assist surgeons in identifying patients at high risk for CLNM, thereby enabling more aggressive surgical strategies when necessary. Concurrently, this model can help avoid unnecessary prophylactic central lymph node dissection in low-risk patients, significantly reducing potential surgical complications such as recurrent laryngeal nerve injury and hypoparathyroidism. This aligns with ongoing efforts to refine personalized surgical planning for PTC patients, including those with lateral lymph node metastasis.

Despite its valuable contributions, this study has several limitations. First, as a single-center retrospective study, it may be subject to selection bias. Consequently, the results require further validation through prospective, multi-center studies to confirm their generalizability across diverse patient populations. Second, this study did not analyze the relationship between dynamic changes in serum Tg and TgAb and CLNM. Further research should explore whether dynamic changes in these markers are associated with CLNM, potentially offering additional prognostic insights. Finally, this study did not integrate molecular markers, such as the BRAF V600E mutation, which are increasingly recognized for their role in PTC aggressiveness and prognosis. Future research could enhance this serological-clinical model by combining it with molecular subtyping to construct a more powerful and comprehensive predictive system for CLNM in PTC.

## Conclusion

In summary, preoperative serum Tg and TgAb, as well as the derived log10 (Tg/TgAb+1), are independent predictors of CLNM in patients with cN0 PTC. They demonstrate good predictive performance for CLNM and may serve as effective and convenient novel markers for predicting CLNM. This provides surgeons with promising adjuncts for preoperative risk stratification, enabling them to formulate individualized surgical plans, such as deciding whether to perform pCND, thereby advancing precision surgical treatment for cN0 PTC patients.

## Data Availability

The raw data supporting the conclusions of this article will be made available by the authors, without undue reservation.
